# Virulence phenotypes of low-passage clinical isolates of Nontypeable *Haemophilus influenzae *assessed using the *chinchilla laniger *model of otitis media

**DOI:** 10.1186/1471-2180-7-56

**Published:** 2007-06-14

**Authors:** Farrel J Buchinsky, Michael L Forbes, Jay D Hayes, Kai Shen, Suzanne Ezzo, James Compliment, Justin Hogg, N Luisa Hiller, Fen Ze Hu, J Christopher Post, Garth D Ehrlich

**Affiliations:** 1 Center for Genomic Sciences, Allegheny-Singer Research Institute/Allegheny General Hospital, 320 East North Avenue, Pittsburgh, Pennsylvania, 15212, USA; 2 Division of Pediatric Otolaryngology, Department of Surgery, Allegheny General Hospital, Pittsburgh, Pennsylvania, 15212, USA; 3 Department of Microbiology and Immunology, Drexel University College of Medicine, Pittsburgh, Pennsylvania, 15212, USA; 4 Division of Pediatric Critical Care & Hospitalist Medicine, Department of Pediatrics, Allegheny General Hospital, Pittsburgh, Pennsylvania, 15212, USA; 5 Department of Animal Husbandry, Allegheny Singer Research Institute/Allegheny General Hospital, Pittsburgh, Pennsylvania, 15212, USA

## Abstract

**Background:**

The nontypeable Haemophilus influenzae (NTHi) are associated with a spectrum of respiratory mucosal infections including: acute otitis media (AOM); chronic otitis media with effusion (COME); otorrhea; locally invasive diseases such as mastoiditis; as well as a range of systemic disease states, suggesting a wide range of virulence phenotypes. Genomic studies have demonstrated that each clinical strain contains a unique genic distribution from a population-based supragenome, the distributed genome hypothesis. These diverse clinical and genotypic findings suggest that each NTHi strain possesses a unique set of virulence factors that contributes to the course of the disease.

**Results:**

The local and systemic virulence patterns of ten genomically characterized low-passage clinical NTHi strains (PittAA – PittJJ) obtained from children with COME or otorrhea were stratified using the chinchilla model of otitis media (OM). Each isolate was used to bilaterally inoculate six animals and thereafter clinical assessments were carried out daily for 8 days by blinded observers. There was no statistical difference in the time it took for any of the 10 NTHi strains to induce otologic (local) disease with respect to any or all of the other strains, however the differences in time to maximal local disease and the severity of local disease were both significant between the strains. Parameters of systemic disease indicated that the strains were not all equivalent: time to development of the systemic disease, maximal systemic scores and mortality were all statistically different among the strains. PittGG induced 100% mortality while PittBB, PittCC, and PittEE produced no mortality. Overall Pitt GG, PittII, and Pitt FF produced the most rapid and most severe local and systemic disease. A post hoc determination of the clinical origins of the 10 NTHi strains revealed that these three strains were of otorrheic origin, whereas the other 7 were from patients with COME.

**Conclusion:**

Collectively these data suggest that the chinchilla OM model is useful for discriminating between otorrheic and COME NTHi strains as to their disease-producing potential in humans, and combined with whole genome analyses, point the way towards identifying classes of virulence genes.

## Background

*Haemophilus influenzae *is a gram-negative coccobacillus that is an obligate resident of the human respiratory mucosa [1,2]. Numerous studies have suggested that *H. influenzae*, present in the nasopharynx of the majority of children and adults [3-5], is a common cause of superinfection following upper respiratory viral infections [6-8]. The *H. influenzae *display multiple pathogenic instruments, including redundant heme acquisition mechanisms, IgA proteases, direct invasion of host columnar epithelial cells, and the release of a host of adhesins and proteins capable of functionally impairing the mucociliary escalator [7,9-11]. The presence of a polysaccharide capsule by some strains has provided for the division of the species into typeable (serotypes, a-f) and nontypeable (NTHi) isolates [12]. The NTHi are frequently recovered from otitis media (OM) effusions and from the sputum and lung biopsy specimens of patients with cystic fibrosis, chronic bronchitis, and chronic obstructive pulmonary disease. Moreover, direct cellular invasion by NTHi may play a role in chronic sinopulmonary infections. *H. influenzae *type b (Hib), prior to the introduction of highly efficacious conjugate vaccines in the late 1980's, was responsible for 95% of the invasive disease associated with this species [13,14]. Emerging evidence has linked the NTHi to invasive disease suggesting that some of the NTHi strains may be evolving to fill the niche previously occupied by Hib [15]. In this study of twenty invasive NTHi strains, 12 of which were from adult or adolescent patients, 18 distinct sequence types were identified indicating that this is not a clonal phenomenon.

Clinical phenotyping studies indicate that there is a broad range of disease symptoms that can be triggered by the NTHi, however the genetics of the virulence mechanisms underlying these myriad phenotypes are just starting to be elucidated [16-21]. Like all infectious processes, NTHi disease results from a set of complicated host-pathogen interactions [22,23], however, in the case of the NTHi this is compounded by their documented genomic heterogeneity [7,24,25]. The laboratory strain Rd was the first free-living organism to have its genome sequenced [26], however NTHi diversity studies have demonstrated that each clinical isolate is genomically unique [2,24,25]. This diversity triggered the development of the distributed genome hypothesis [25,27,28] which posits that at the population level there is a supragenome which is multiple times the size of the genome of any single bacterium, and that each strain contains a unique subset of the contingency genes that make up the supragenome. This high degree of genomic plasticity among strains, polyclonal nasopharyngeal colonizations [29], and the possession of autocompetence and autotransformation mechanisms provide the NTHi collectively with the ability to continually generate new forms, some of which will have novel combinations of virulence traits.

The NTHi are responsible for greater than 30% of all OM cases which is the most frequent complaint for emergency department and primary care physician visits by children less than 16 years of age worldwide – accounting for some 25,000,000 annual physician encounters [30]. Moreover, OM can be complicated by direct invasion into adjacent organs causing mastoiditis and/or meningitis [31,32]. An understanding of the genetic repertoire of the NTHi associated with OM and its complications will aid in the development of prevention and treatment strategies ranging from vaccines to antimicrobials [6,25,33].

In the current study we have used the chinchilla (*Chinchilla laniger*) model of OM to investigate differences in the clinical phenotypes of 10 NTHi strains obtained from patients with chronic OM with effusion and otorrhea. Studies at our institution and others have demonstrated the applicability of the chinchilla model of OM to human disease as it provides an inexpensive, reproducible middle-ear infection in nearly 100% of inoculated animals that has yielded numerous insights into the molecular pathophysiology and microbiology of mammalian middle ear disease [34-41].

## Results

### Differences in rapidity and severity of otologic signs

**Figure 1:**
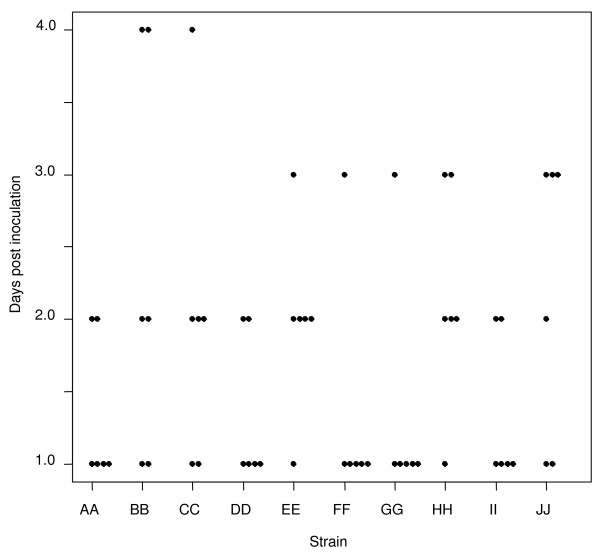
**Rapidity of local disease onset**. Scatterplot showing the number of days it took for chinchillas inoculated with the 10 clinical NTHi strains to develop moderate or worse (score =/> 2) local (otologic) disease. X-axis = the clinical NTHi strains (PittAA-PittJJ, left to right); Y-axis = the days post inoculation that moderate or worse local disease developed.

**Figure 2:**
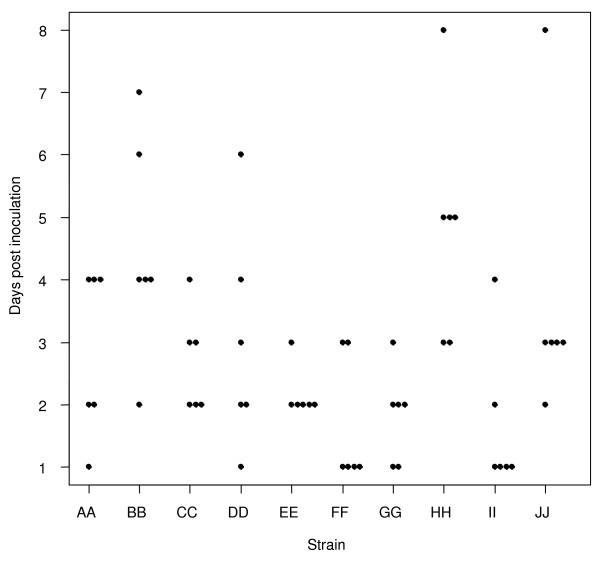
**Rapidity of most severe local disease**. Scatterplot showing the number of days it took for chinchillas inoculated with the 10 clinical NTHi strains to develop their maximum (most severe) otologic score – regardless of what that score was. X-axis = the clinical NTHi strains (PittAA-PittJJ, left to right); Y-axis = the days post inoculation that moderate or worse local disease developed.

**Figure 3:**
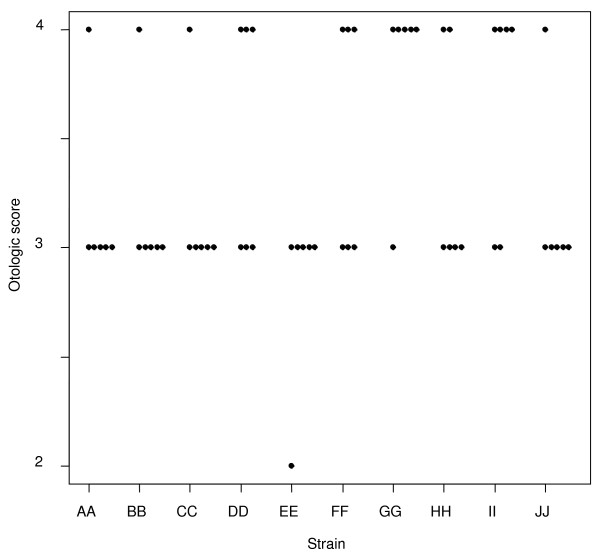
**Maximum otologic score per animal**. Scatterplot showing the maximum otologic severity – regardless of time – recorded for each of the chinchillas inoculated with the 10 clinical NTHi strains. X-axis = the clinical NTHi strains (PittAA-PittJJ, left to right); Y-axis = the otologic clinical score based upon the criteria in Table 1.

The first criterion we evaluated was days to the development of unambiguous otologic signs (local disease) which we defined as a score of 2 or higher based upon the otologists comments that the difference between 0 and 1 (Table 1) was often difficult to discern. Using this criterion there was no statistical difference among the strains with respect to rapidity of local disease onset (Figure 1). However, the variability in the amount of time for each strain to induce its maximum otologic scores (i.e. how many days following infection did it take before the animals exhibited their most severe local signs) was highly statistically significant; an ANOVA analysis of these data produced a p value = 0.00087. A scatterplot of these data indicates that strains PittFF, PittGG, and PittII demonstrated the most rapid onset of severe local signs (Figure 2). Moreover the differences in the mean maximal otologic scores among the 10 strains were significantly different for each of the first five days following infection, after which the high mortality rates for the more systemically virulent strains made such analyses problematic given the missing data (Table 2). Similarly, it was determined that the mean maximal otologic score per animal, regardless of day on which it was recorded, induced by each of the 10 NTHi strains was statistically significant; ANOVA analysis produced a p value = 0.022 (Figure 3).

**Table 1:** Scoring system to quantify Haemophilus influenza [NTHi] pathogenicity in the CGS-chinchilla model.

Otologic Score	0	1	2	3	4	
Degree of otoscopic changes (inflammation)	None	Mild	Moderate	Frank purulence	Tympanic membrane rupture	

Systemic Score	0	0.5	1	2	3	4

Systemic description	normal	Slightly lethargic Upright and steady on feet Immediately responds to stimulation by actively moving around cage Eating and drinking	Slightly lethargic Upright and steady on feet Head and ears down Responds by actively moving around cage rapidly when stimulated by voice or touch Eating and drinking Eyes 1/2 open	Moderately lethargic Slightly ataxic Able to keep self upright Moving around cage slowly only when stimulated by voice or touch Eating treats and drinking water	Same as 2 but not eating or drinking Supportive treatment given (fluids and Buprenex)	Extremely lethargic Extremely ataxic Not able to maintain an upright position Barely moving around cage Dyspnea Sacrificed

**Table 2:** Statistical significance of the mean maximum differences in otologic scores induced by 10 clinical NTHi strains (PittAA – PittJJ) following transbullar inoculation into chinchillas.

Days Post Inoculation	p value
Day 1	0.0005825
Day 2	0.003254
Day 3	0.000005038
Day 4	0.004122
Day 5	0.02772*

* Due to extensive mortality beginning on day 5 associated with the more systemically virulent strains, there are many data points missing which artificially lowers the p value as evaluations could not be performed on dead animals.

### Differences in rapidity and severity of systemic signs

**Figure 4:**
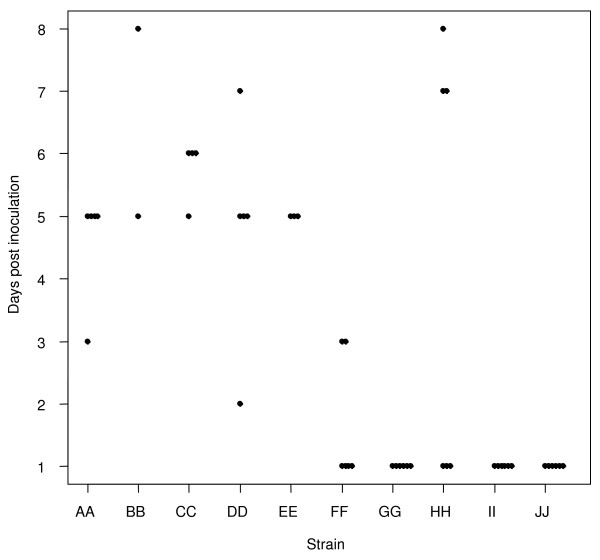
**Rapidity of systemic disease onset**. Scatterplot showing the number of days it took for chinchillas inoculated with the 10 clinical NTHi strains to first develop their maximum (most severe)significant systemic signs (systemic score ≥1 – regardless of what their eventual maximum severity was. X-axis = the clinical NTHi strains (PittAA-PittJJ, left to right); Y-axis = the days post inoculation that the first signs of systemic disease developed.

**Figure 5:**
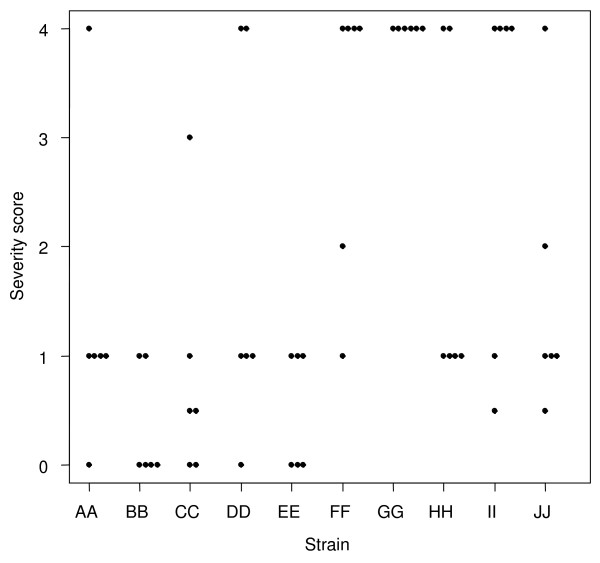
**Maximum systemic severity**. Scatterplot showing the maximum systemic severity – regardless of time – recorded for each of the chinchillas inoculated with the 10 clinical NTHi strains. X-axis = the clinical NTHi strains (PittAA-PittJJ, left to right); Y-axis = the systemic clinical score based upon the criteria in Table

An ANOVA analysis of the differences among the strains with respect to the rapidity of the development of the first signs of systemic disease produced extremely highly significant results: p = 2.5 × 10^-7^. However, this figure actually underestimates the real differences among the strains as animals that never developed systemic signs were excluded from the analysis. A scatterplot of these data demonstrate that four, three, two, one, and one of the animals inoculated with strains PittBB, EE, CC, AA, and DD, respectively, never evidenced any systematic signs and are therefore not plotted in Figure 4. This figure also shows that PittGG, II, and JJ induced systemic signs in 100% of the animals within 24 hours. The maximum severity score per animal is detailed in Figure 5 and ANOVA demonstrated that the ten strains did not induce equivalent maximum systemic severity (p = 2.2 × 10^-5^.). There was weak correlation between rapidity of onset of systemic illness and the maximal severity of that illness in any animal (Spearman's rank correlation rho = -0.36 with p = 0.011). Finally we determined that there was a weak correlation between otologic and systemic severity in the animals (Spearman's rank correlation rho = 0.28 with p = 0.029)

### Differences in mortality

**Figure 6:**
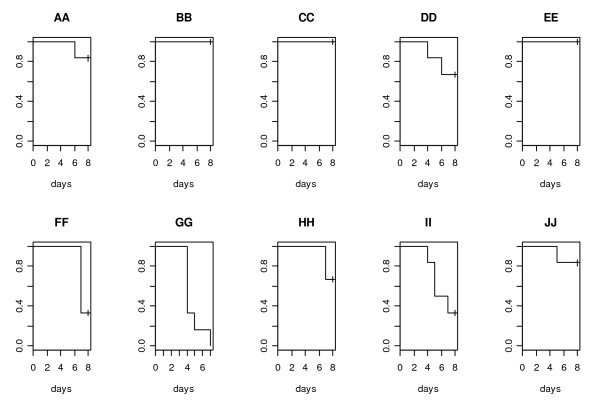
**Differences in mortality**. Kaplan-Meyer plots showing the differences in mortality induced by the 10 NTHi strains, PittAA-PittJJ. X-axis = time in days following inoculation; Y-axis percentage of surviving animals.

The mortality of the chinchillas was highly correlated with the inoculating strains. All of the animals infected with PittGG succumbed, and two thirds of the animals infected with PittFF and PittII also died, whereas none of the animals infected with PittBB, PittCC, or PittEE died (Table 3). A Fisher's exact test for these count data yielded a highly significant p-value = 0.00019, and Kaplan-Meyer plots of these data indicate the increased rapidity and percentages of death associated with the PittGG, PittII and PittFF compared with the other seven strains. (Figure 6). A complete analysis of strain pairwise comparisons for differences in mortality demonstrated that PittGG was statistically more likely to be associated with death than PittBB, or PittCC, or PittEE (p-values = 0.0039), or PittAA and PittJJ (p-values = 0.019). However, after the application of a Bonferroni correction none of these values reached statistical significance due to the relatively small cohort sizes and the large number of tests performed (n = 45).

**Table 3:** Survival and mortality of chinchillas inoculated with 10 different clinical NTHi strains

**Strain**	PittAA	PittBB	PittCC	PittDD	PittEE	PittFF	PittGG	PittHH	PittII	PittJJ
**Fate**										

Survived	5	6	6	4	6	2	0	4	2	5
Died	1	0	0	2	0	4	6	2	4	1

### PCR-based analyses of specimens

Upon death all of the chinchillas were evaluated for NTHi DNA using a PCR-based assay [40]. Microbial culture was not employed because according to the IACUC protocol all animals that had shown signs of systemic or invasive disease were treated with antibiotics, which we have previously demonstrated in the chinchilla model, will render cultures uniformly negative in spite of active bacterial infections [37]. Middle-ear effusions, or lavages if there was no frank effusion, were recovered and assayed for *H. influenzae *DNA and established that all of the animals had been productively infected including those with minimal local and absent systemic signs.

### Clinical origins of the NTHi strains

After completion of the animal studies and the statistical analyses, a *post hoc *inquiry was made to determine the exact clinical origins of the 10 NTHi strains under evaluation. Although all strains were derived from pediatric middle-ear specimens, PittGG, PittFF, and PittII, the consistently most virulent strains both otologically and systemically were identified as being otorrheic in origin, having been isolated from children with a perforated tympanic membrane, whereas the other seven were obtained from patients undergoing tympanostomy and tube placement for COME, a less virulent disease.

### Global comparative genomics of the NTHi strains

**Figure 7:**
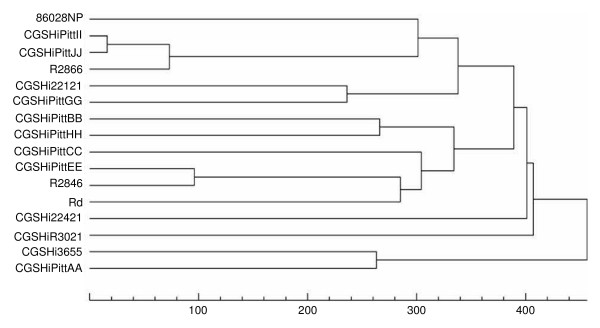
**Degree of genic sharing of distributed or non-core genes**. Dendrogram developed using the unweighted pair group method algorithm demonstrating the degree of genic sharing of distributed or non-core genes [26,28] which are defined as the set of genes not universally present among all strains of the species. The figure compares 15 NTHi strains, which include 9 of the strains phenotyped in the current study (PittDD was omitted due to incomplete genomic data and PittFF and PittGG collapse to a single strain using this method) and the laboratory strain Rd. The sequence for the 86028NP strain has been previously published [18], and the unannotated sequences for the R2866 and R2846 NTHi strains were obtained from Genbank (accession #s NZ_AADP00000000, and NZ_AADO00000000, respectively) and used with permission of the depositing authors. The X-axis lists the number of genic differences between strains; y-axis lists the *H. influenzae *strains. Strain 86028NP is a nasopharyngeal (NP) isolate obtained from a patient suffering from OM; R2866 is an invasive strain; CHSHi22121 is an NP isolate from a well child; R2846 is an COME isolate; CGSHiR3021 and CGSHi22421 are NP isolates from healthy children; and CHSHi3655 is an OME strain.

The entire genomes of nine of the ten NTHi clinical strains (PittDD was excluded due to incompleteness of the genomic data) that were compared with respect to disease phenotype in this study were subjected to global analyses to determine their overall levels of genic relatedness along with seven other clinical NTHi strains, the laboratory strain Rd and a serotype b strain using the unweighted pair group method algorithm (Figure 7). It can be seen at this global level of comparison that strains do not cluster overall by clinical phenotype with the exception of PittGG and PittFF which turned out to be serial isolates from the same patient and do not have any significant genic differences, but only allelic differences. PittGG/FF do not cluster with the other invasive strains including PittII from this study and R2866 from a published study [17]. Similarly the COME strains do not cluster together when observed from a global genomic viewpoint.

## Discussion

This *in vivo *comparative study of the propensity of ten clinical NTHi strains to induce otologic and systemic disease in the chinchilla following transbullar inoculation clearly demonstrates that different strains have varying abilities to cause both local and systemic disease. This is the first demonstration of the utility of chinchilla model of OM to distinguish among multiple NTHi clinical strains with respect to each one's myriad virulence parameters. These findings were not wholly unsuspected in light of the fact that each of these strains has been demonstrated to contain a different subset of distributed genes from the NTHi supragenome [25], nonetheless when you combine the fact that the functions of most of these novel genes are unknown with the observation that the chinchilla is not a natural host of the NTHi it was not obvious either. It is of particular interest that this disease model was able to distinguish, when evaluated in a blinded manner, between strains of COME origin and invasive strains of an otorrheic origin. The otorrheic strains, PittGG, PittFF and PittII, consistently produced the most rapid and severe otologic and systemic signs and produced a combined mortality of 77.8% (14/18), whereas the seven COME strains only had a combined mortality of 14.3% (6/42). Strains HH and JJ, which are of the same MLST type [25], a measure of the relatedness of core metabolic genes, belong to completely different clades when evaluated by shared distributed genomic characters (Figure 7). Interestingly Pitt JJ and PittII, respectively COME and invasive strains, cluster most closely together in terms of shared distributed genes. The fact that both of these strains are associated with rapid systemic disease onset, but that only PittII induces severe systemic disease suggests that careful analyses of these two strains may provide candidate genes associated with systemic severity or limiting systemic severity. PittAA, which is clearly the most genomically unique of the phenotyped strains, produces local and systemic clinical profiles nearly identical with PittBB, CC and EE. Taken together, the disease phenotype data and the global-level comparative genomic data suggest that it is not possible to predict disease phenotype based on simply viewing the degree of overall genic relatedness. This is not a surprising finding as the mean number of genic differences among each of the possible strain pairs is > 350 (data not shown), and the number of genes that are associated with each of the various parameters of clinical virulence is most likely a small fraction of this number. We are pursuing a two-pronged approach to this problem of identifying disease genes associated with specific virulence phenotypes. In the first case once the point is reached where sequencing additional NTHi strains does not materially increase the size of the supragenome we will do quantitative trait loci (QTL)-like genic association studies using an exhaustive distributed genome chip to interrogate hundreds of clinically characterized strains. In the second case we will perform metabolomic reconstructions of multiple strains grouped by virulence phenotype to identify shared pathways.

Little is known of what specific genotypic features are important for the development of local and systemic disease among the NTHi. Erwin et al (2005) in a study of 17 invasive NTHi strains concluded that invasive isolates are genetically and phenotypically diverse, but that some loci are frequently found in association [17]. The systemically virulent PittGG/FF strains examined in the current study contain the *hif *operon which encodes a pilus gene cluster that mediates adherence to sialic acid-containing lactosylceramide structures on epithelial cell surfaces [49]; these genes have previously been implicated as being more prevalent in throat isolates than COME isolates and are nearly ubiquitous among the invasive type b strains [50]. Most NTHi strains associated with chronic disease including PittAA and PittJJ do not contain the *hif *operon, but PittAA contains one of the *hmw *cassettes (HMW1A), however, Pitt JJ is lacking all of the hmw genes as well; which are much more rarely found in invasive strains as these proteins mediate adherence to the respiratory epithelial cells. Similarly the *lic *genes which encode LOS moieties, including phosphoryl choline, have been previously associated with chronic disease, but are often absent from invasive strains [17,51]; among the chronic strains in the current study PittBB and PittEE contained genes from this cluster whereas PittAA and PittJJ did not.

To control for the small sample sizes and minimize bias in the current study we used very conservative statistical analyses and employed a single highly qualified individual to perform all of the otologic evaluations in a blinded manner. In addition we varied daily the order in which the animals were evaluated. The fact that our intra-strain variability was significantly less than our inter-strain variability strongly suggests that the model performed as expected. These findings together with the model's ability to differentiate the otorrheic strains from the COME strains and its phenotypic clustering of PittFF and PittGG which we subsequently determined were by far the most similar strain-pair genotypically augers well for the continued use of this model for characterizing NTHi clinical strains.

## Conclusion

This study provides validation of the discriminatory power of the chinchilla-NTHi-induced otitis media model for use as a tool in stratifying disease phenotypes of COME- and otorrhea-derived NTHi-isolates. In the current study, significant differences were demonstrated both with respect to local and systemic virulence parameters among a set of 10 low-passage clinical NTHi isolates. Moreover, the three strains that induced the most rapid and severe systemic disease, as well as the most rapid onset of local disease were determined *post hoc *to have been isolated from a more clinically aggressive disease condition. We will be performing whole genome evaluations of these strains using an NTHi supragenome chip composed of all identifiable NTHi genes derived from a project to sequence 40 geographically and clinically diverse NTHi strains in their entirety.

Identification of the genetic bases for the various virulence phenotypes including chronic persistence, local invasiveness, and systemic illness will have important implications for vaccine and antimicrobial development. With increasing knowledge of the diversity of the individual NTHi genomes [2,17,25,29,52-54], and the size of the NTHi supragenome [25,27], additional studies of phenotypic diversity will be needed to clarify the role of individual genic elements as well as various combinations of discrete genic elements. It is becoming clear that NTHi recovered from disease states are genotypically different from those harvested from carrier states [17,18,54]. Conceivably, genetic heterogeneity among the NTHi combined with auto-competence processes may provide the necessary genetic reservoir and the means for the development of novel virulent strains *in vivo *via recombination between commensal residents and infecting pathogens.

## Methods

### Bacterial strains and culture

Ten NTHi strains (PittAA-PittJJ) were obtained through the Clinical Microbiology Laboratory of Children's Hospital of Pittsburgh that had been isolated from children with either chronic otitis media with effusion (COME) or otorrhea [25]. All strains were cultured in brain heart infusion broth (Becton Dickinson, Sparks, MD) supplemented with 10 μg/ml hemin (Fisher Scientific, Pittsburgh, PA), 2 μg/ml NAD (Sigma, St. Louis, MO) and 20-μg/mL thiamine HCl (Sigma), and grown at 37°C in a humidified 5% CO_2 _environment. All isolates were received as first plate specimens on chocolate agar. Each strain was grown up once in supplemented BHI from a single colony picked from the chocolate agar plate to mid-log phase and then used to make a large number of freezes using a 1:1 mixture of a glycerol salts solution (40 mM KCl, 40 mM NaCl, 1 mM MgSO_4_, 65% glycerol v/v). For subsequent cultures a scraping from one of the still frozen glycerol freezes was inoculated directly into supplemented BHI. Upon sequencing the clinical strains PittFF and PittGG, which was accomplished subsequent to our phenotypic characterizations and after the first draft of this paper was written, we determined that they contained essentially the same genomic content, i.e. there were no genic differences between the two strains, only some allelic differences that have not been confirmed. Thus, a more detailed analysis of their origins was conducted that revealed they were sequential otorrheic isolates from the same patient most likely made on consecutive days. The COME strains were obtained at the time of myringotomy and tube placement and the otorrheic strains were isolated from drainage through a perforated tympanic membrane. The Allegheny County Public Health Laboratory and the New York State Department of Health's Wadsworth Laboratory identified all 10 strains as nontypeable [25] and these findings were confirmed using a PCR-based capsular typing methodology [24,42,43].

### Induction of OM in the chinchilla and experimental design

All experiments were conducted with the approval of the Allegheny Singer Research Institute's Institutional Animal Care and Use Committee (IACUC). Young adult chinchillas (*C. laniger*, 400–600 gm; McClenahan Chinchilla Ranch, New Wilmington, PA) were obtained free of middle-ear disease as culls from the fur industry. After a 7 day acclimation period, the animals were anesthetized on experimental day 0 by intramuscular injection of 0.1 ml of a solution of ketamine hydrochloride 100 mg/ml, xylazine hydrochloride 30 mg/ml and acepromazine 5 mg/ml. After anesthesia was confirmed (abolishment of eye-blink reflex), 0.1 ml of a 10^5 ^colony forming units (CFU)/ml NTHi culture was injected bilaterally through the tympanic bullae using a 0.5 in, 27-gauge needle on a 1 ml syringe. Each of the 10 strains was used to infect six chinchillas.

Animals were monitored daily for seven days for signs and severity of local (otologic) and systemic disease using the criteria in Table 1. All evaluations were performed by observers who were blinded with regard to the inoculating strains. Local disease was evaluated via a single otoscopist [JCP] to ensure uniformity. Hence for each animal three scores were recorded: otoscopy score for right ear, otoscopy score for left ear and systemic score. From the collected data we determined measures relating to rapidity of local disease: 1) days to first significant otologic score; and 2) days to maximum otologic score. Systemic evaluations included rapidity of onset, maximum severity of disease, and mortality.

Following sacrifice *H. influenzae*-specific PCR [40] was performed on autopsy specimens to verify the causative organism as NTHi.

### Statistical analyses

All analysis was performed by using the scores that were recorded daily for each ear and for each animal in the case of the systemic severity. The data was not transformed. Nevertheless, for each animal, the maximum of the left and right ear score was used. Thus the data were considered independent at the level of each animal. Statistical analysis was performed using R: A Language and Environment for Statistical Computing (R Development Core Team, Vienna, Austria, 2006) [44]. For each parameter referred to in the text, one-way analysis of variance (ANOVA) was performed with the null hypothesis being that the parameter was equal amongst all 10 strains of infecting bacteria. Significance was defined as p < 0.05. Repetition of the analysis by the non-parametric Kruskal-Wallis test yielded similar results. When the null hypothesis was rejected Tukey multiple comparisons of means test was performed so that the mean of each strain could be compared to every other strain.

Mortality was primarily evaluated by determining the number of animals that were sacrificed prematurely by each infecting strain. The 10 strains were compared by the Fisher's Exact Test for Count Data in which the null hypothesis was that the number of animals dying prematurely was equal amongst all strains. Pairwise comparison of proportions was used to explore the premature mortality of each strain in contrast to the each of the others. Since the sample size for each strain was small, and the Bonferroni correction was used, the null hypothesis could not be rejected for any specific pairing.

### Global genic comparisons and gene possession-based phylogenetic tree building among *H. influenzae *strains

Each of the *H. influenzae *clinical strains that were evaluated for disease phenotype in the chinchilla OM model was also subjected to whole genome sequencing using the 454 LifeSciences pyrosequencing technology. In addition, several additional *H. influenzae *clinical strains were sequenced and included in the analysis to provide a perspective on the relative relatedness of the strains in this analysis compared to the overall species-level diversity. Gap closure experiments to join assembled contigs were designed by a custom Perl script, and PCR primers were designed by Primer3 [45]. Coding sequences for all 17 strains in the analysis, including those previously annotated, were identified by the AMIgene microbial gene finder adjusted to low-GC parameters and trained on the Rd KW-20 genome to ensure that all subsequent gene cluster analyses began with a common annotation [46]. Each pair of genes within each genome and among all genomes was examined for protein homology by alignment of six-frame nucleotide translations to predicted protein sequences. Alignments were generated by tfasty34, part of the Fasta v3.4 package [47]. Six-frame translations were employed to minimize the impact of frame-shift artifacts. Each gene was also aligned against the full nucleotide sequence of the 17 genomes by fasta34. Genes were clustered based on homology using a single-linkage algorithm. A link was defined by a significant tfasty match between genes which exceeded an identity threshold of 70% and covered at least 70% of the shorter gene. The asymmetric length criterion was chosen to insure that any fragmented genes would cluster with the full length version of the gene. A side-effect of this criterion is that multi-domain proteins will fuse with proteins which are composed of a subset of those domains. Significant fasta matches between genes and genomic sequence were used to identify sequence conservation between a gene cluster and a strain. A gene possession-based phylogenetic tree of the 17 strains was constructed by defining the distance between a pair of genomes i and k to be ∑n|gn,i−gn,k|
 MathType@MTEF@5@5@+=feaafiart1ev1aaatCvAUfKttLearuWrP9MDH5MBPbIqV92AaeXatLxBI9gBaebbnrfifHhDYfgasaacH8akY=wiFfYdH8Gipec8Eeeu0xXdbba9frFj0=OqFfea0dXdd9vqai=hGuQ8kuc9pgc9s8qqaq=dirpe0xb9q8qiLsFr0=vr0=vr0dc8meaabaqaciaacaGaaeqabaqabeGadaaakeaadaaeqbqaamaaemaabaGaem4zaC2aaSbaaSqaaiabd6gaUjabcYcaSiabdMgaPbqabaGccqGHsislcqWGNbWzdaWgaaWcbaGaemOBa4MaeiilaWIaem4AaSgabeaaaOGaay5bSlaawIa7aaWcbaGaemOBa4gabeqdcqGHris5aaaa@3EA1@ where gn, i = 1 if gene n is present in strain i and 0 otherwise. The strains were clustered based on the distance metric by the unweighted group average method implemented in clustering utilities [48] which results in a tree that shows relationships based upon the number of non-core genes that each strain pair has in common. Non-core genes are defined as genes that are present in only a subset of strains, as opposed to core genes which are universally present among all strains of a species. The grouping of gene clusters into core and non-core genes simply reflects the distribution of each cluster relative to all sequenced strains. The genomic sequences of the NTHi clinical strains that were evaluated phenotypically in this study are in the process of being submitted to Genbank as part of a separate study (Hogg et al submitted).

## Authors' contributions

All authors have given final approval of this manuscript.

FJB designed and performed all of the statistical analyses and co-wrote the revised version of the manuscript.

MFL prepared the first draft of the manuscript and assisted with study design and data collection.

JDH, KS, SE, JC, JH and NLH made substantial contributions to the acquisition, and analysis of the data.

FZH participated in the design and supervised all of the laboratory studies.

JCP participated in its design, developed the phenotypic scoring system, and performed all of the otologic examinations.

GDE conceived of the study; participated in its design; assisted with all of the animal laboratory studies; oversaw all aspects of the data analysis and co-wrote the revised version of the manuscript.
